# PPAR*δ* as a Metabolic Initiator of Mammary Neoplasia and Immune Tolerance

**DOI:** 10.1155/2016/3082340

**Published:** 2016-12-18

**Authors:** Robert I. Glazer

**Affiliations:** Department of Oncology, Georgetown University Medical Center and the Lombardi Comprehensive Cancer Center, 3970 Reservoir Rd, NW, Washington, DC 20007, USA

## Abstract

PPAR*δ* is a ligand-activated nuclear receptor that regulates the transcription of genes associated with proliferation, metabolism, inflammation, and immunity. Within this transcription factor family, PPAR*δ* is unique in that it initiates oncogenesis in a metabolic and tissue-specific context, especially in mammary epithelium, and can regulate autoimmunity in some tissues. This review discusses its role in these processes and how it ultimately impacts breast cancer.

## 1. Introduction

The PPAR nuclear receptor family consists of the PPAR*α*, PPAR*γ*, and PPAR*δ*/*β* isotypes, which function as heterodimeric partners with RXR with specificity dictated by high-affinity binding of PPAR ligands and coactivators [1]. Similar to other nuclear receptors, PPARs contain an N-terminal transactivation domain, a DNA-binding domain, a ligand-binding domain, and a C-terminal ligand-dependent transactivation region [2]. PPARs bind to a DR-1 response element (PPRE) with the consensus sequence AGG(T/A)CA that is recognized specifically by the PPAR heterodimeric partner [3]. Ligand-activated PPARs interact with coactivators CEBPA/B and NCOA3 and in the unliganded state with corepressor NCOR2 [4–7]. Of the three isotypes, PPAR*δ* plays a dominant role in regulating fatty acid *β*-oxidation, glucose utilization, cholesterol transport, and energy balance [8–10] but also modulates the cell cycle, apoptosis, angiogenesis, inflammation, and cell lineage specification [11–14]. These multifaceted functions indicate that PPAR*δ* has a critical homeostatic role in normal physiology and that its aberrant expression can impact the initiation and promotion of oncogenesis. This review discusses recent advances pertaining to the involvement of PPAR*δ* in these processes primarily as they relate to mammary tumorigenesis.

## 2. PPAR*δ* and Tumorigenesis

The role of PPAR*δ* in tumorigenesis has been investigated for almost two decades, and whether it exerts an oncogenic or antioncogenic role depends in large part on the targeted tissue and the gene targeting strategy utilized [14–16]. In the context of the mammary gland, however, most animal models confirm that PPAR*δ* exerts an oncogenic effect. This can be envisioned to result in part from competition between the tumor promoting effects of PPAR*δ* and the tumor suppressor effects of PPAR*γ*. PPAR*γ* agonists reduce mammary carcinogenesis [17–19], which correlates with induction of PTEN [20, 21] and BRCA1 [22] tumor suppressor activity, as well as reduction of inflammation via the Cox2/Ptgs2 pathway [23]. Conversely, PPAR*γ* haploinsufficiency [23] or expression of a dominant-negative Pax8-PPAR*γ* transgene [24] and direct or indirect inhibition of PPAR*γ* [21, 25] enhance DMBA mammary carcinogenesis. In MMTV-Pax8-PPAR*γ* mice, the increased rate of carcinogenesis correlates with enhanced Wnt, Ras/Erk, and PDK1/Akt signaling, reduced PTEN expression, and a more stem cell-like phenotype [24]. The respective Yin/Yang functions of PPAR*δ* and PPAR*γ* are consistent with the ability of PPAR*δ* to enhance survival through the PI3K and PDK1 pathways in response to wound healing [26, 27], as well as with the proliferative and angiogenic response of breast cancer and endothelial cells to conditional activation of PPAR*δ* [28]. The induction of PDK1 signaling by the PPAR*δ* agonist GW501516 in DMBA-treated wild-type mice [19], the increased expression of PPAR*δ* in GW501516-treated MMTV-PDK1 mice [29], and reduction of mammary tumorigenesis in MMTV-Cox2 mice crossed into a PPAR*δ* null background [30] further support its oncogenic potential. This outcome was ultimately proven by the generation of MMTV-PPAR*δ* mice, which developed infiltrating mammary adenocarcinomas and whose progression was accelerated by, but not dependent on, agonist stimulation [31]. From a clinical perspective, this result is concordant with the increased expression of PPAR*δ* in invasive breast cancer [12, 32] and by manifestation of a PPAR*δ* signaling network that predicts poor survival in this disease [33].

**Figure 1:**
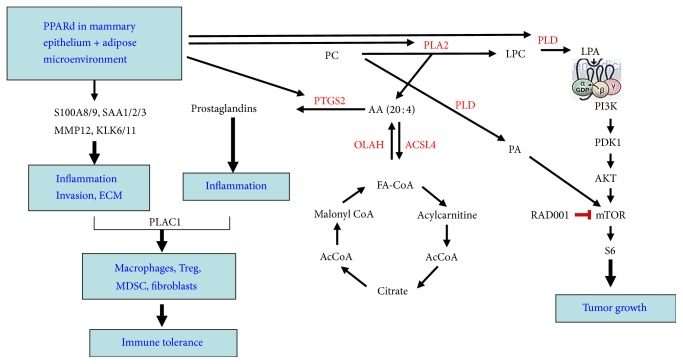
Interactions between inflammation, metabolism, and mTOR signaling in the mammary gland of MMTV-PPAR*δ* mice. PPAR*δ* activates PPRE-containing genes associated with metabolism (Olah, Ptgs2, Pla2, and Pld), invasion (Mmp12, Klk6), and inflammation (S100a8/9, Saa1/2/3). Arachidonic acid (AA) is a substrate for Ptgs2 and is a constituent of phosphatidylcholine (PC) required for prostaglandin synthesis. Lysophosphatidylcholine (LPC) is generated from PC by phospholipase A2 (Pla2), and lysophosphatidic acid (LPA) and phosphatidic acid (PA) are generated by phospholipase D (Pld). LPA stimulates mTOR through a G protein-coupled receptor, and PA directly activates mTOR. The mTOR inhibitor RAD001 (everolimus) inhibits tumorigenesis in this animal model. The net result is an increase in inflammation, extracellular matrix remodeling, immune suppression, and neoplasia. Adapted from [31].

A signature feature of MMTV-PPAR*δ* mice is the development of ER^+^/PR^+^/ErbB2^−^ tumors resembling the luminal B subtype of breast cancer [31], which is denoted by lower ER expression, higher Ki-67 staining, and a higher histologic grade [34]. Since ER mRNA is relatively low in these mice in comparison to immunohistochemical staining, it suggests that PPAR*δ* may affect ER stability posttranslationally, for example, phosphorylation of ER Ser167 by mTOR/S6K [35], a pathway activated in this mouse model (Figure 1). The development of ER^+^ tumors in MMTV-PPAR*δ* mice is similar to what was observed in DMBA-treated MMTV-Pax8-PPAR*γ* mice [24] and DMBA-treated wild-type mice administered the irreversible PPAR*γ* inhibitor, GW9662 [25]. These findings support the notion that PPAR*γ* and PPAR*δ*, either by direct competition [36], cofactor competition [37], and/or ligand-dependent activation [38] have opposing actions that affect expansion of the ER^+^ lineage tumor subtype. Interestingly, ER^+^ tumors also arose in MMTV-NCOA3 mice [39, 40], but not in other MMTV-driven transgenic models [41], suggesting that it is the PPAR*δ* coactivator complex itself, rather than the MMTV promoter that drives expansion of the ER^+^ lineage. This conclusion is also supported by the similarities between MMTV-NCOA3 and MMTV-PPAR*δ* mice for activation of the mTOR signaling axis [39, 40], suggesting its importance in ER^+^ luminal tumor specification.

Another intriguing feature of MMTV-PPAR*δ* mice is the association between the onset of neoplasia and the upregulation of Plac1 [31], a microvillous membrane protein expressed primarily in trophoblasts, but not in most somatic tissues [42] (Figure 1). Plac1 is reexpressed in several malignancies [43–45], and reduction of Plac1 in breast cancer cells inhibits proliferation and invasion [43]. These findings suggest that Plac1 may serve as a diagnostic biomarker as shown by the more favorable prognosis of colorectal cancer patients expressing Plac1 autoantibodies [46]. Analysis of a limited set of paired breast cancer specimens indicates that Plac1 expression is elevated in the majority of biopsies, but not in adjacent normal tissue (Isaacs and Glazer, unpublished results), which is consistent with the presence of circulating Plac1 RNA in the majority of breast cancer subjects [43, 44]. The high level of expression of Plac1 in MMTV-PPAR*δ* mice also suggests that Plac1 may be under the transcriptional control of PPAR*δ* as demonstrated by its dependence on the PPAR*δ* coactivators CEBPA and CEBPB [47] and the presence of PPREs in the promoter regions of mouse and human* Plac1 *(http://www.genecards.org/cgi-bin/carddisp.pl?gene=PLAC1&keywords=plac1).

## 3. PPAR*δ* and Inflammation

One of the earliest recognized functions of PPAR*δ* was its antiapoptotic, chemotactic, and inflammatory actions mediated through the Akt and Rho pathways in response to wound healing in keratinocytes [26, 27, 48]. This was the first indication that PPAR*δ* might be a contributing factor to inflammatory disorders, such as psoriasis [49], and tumorigenesis. It had been previously shown that inflammatory molecules, such as eicosanoids, could serve as endogenous PPAR*δ* ligands [50–52]. In colon tumorigenesis and colitis, Ptgs2 and prostaglandin synthesis are dependent on PPAR*δ* [53, 54], whereas inhibition of tumorigenesis by NSAIDs results from induction of the endogenous PPAR*δ* antagonist, 13-S-hydroxyoctadecadienoic acid [55]. Of note is that a similar Ptgs2/prostaglandin phenotype is expressed in MMTV-PPAR*δ* mice (Figure 1) [31], which is consistent with the induction of mammary tumorigenesis in MMTV-Ptgs2 mice [56], but not in PPAR*δ*-null mice [30]. These findings suggest a feed-forward mechanism, whereby transactivation of the prostaglandin E2 receptor, Ptger4, by PPAR*δ* [57], coupled with the generation of arachidonic acid by phospholipase A2 [58] and the biosynthesis of prostaglandin E_2_ (PGE_2_) via Pges2, elicits a self-sustaining inflammatory response.

In addition to activation of the prostaglandin axis, PPAR*δ* increases expression of the acute phase proteins Saa1, Saa2, S100a8, and S100a9, as well as several members of the kallikrein gene family [31], all of which are elevated in ER^+^ breast cancer [59, 60] and whose promoter regions contain PPREs. S100a8 and S100a9 are ligands for Ager (advanced glycation end-product receptor), another PPAR-dependent gene that mediates acute and chronic inflammation, tumor development, and metastasis in several types of cancer and proliferative disorders [61, 62], including gastric carcinogenesis [63] and psoriasis [49]. Thus, there is strong evidence to implicate PPAR*δ* in driving multiple inflammatory pathways implicated in tumorigenesis.

## 4. PPAR*δ* and Metabolism

PPAR*δ* is one of the primary regulators of intermediary metabolism, including fatty acid synthesis and *β*-oxidation, particularly in adipose and muscle tissue [13, 64]. In MMTV-PPAR*δ* mice, PPAR*δ* functions as an integrator of metabolism and tumorigenesis via the biosynthesis of lysophosphatidic acid (LPA), a metabolite that promotes mammary tumorigenesis [65, 66], and phosphatidic acid (PA), a metabolite that directly activates mTOR [67] (Figure 1). The LPA/PA signaling pathway is also coupled to expression of Pdk4, a PPAR*δ*-regulated inhibitor of pyruvate oxidation that increases unsaturated fatty acid, arachidonic acid, LPA, and PA biosynthesis in MMTV-PDK1 mice [29, 31] and is in accordance with the capacity of long chain unsaturated fatty acids to serve as endogenous PPAR*δ* ligands [50–52]. Additionally, PPAR*δ* upregulates the fatty acid-binding protein (FABP) gene family [68], which facilitate fatty acid transport and potentiate EGFR- and ErbB2-mediated proliferation [69, 70] and invasion [71]. Lastly, PPAR*δ* and fatty acid oxidation are required to maintain asymmetric stem cell division [72], an area that may be linked to ER^+^ tumor specification and one unexplored thus far in mammary tumorigenesis.

## 5. PPARs and Immune Tolerance

**Figure 2:**
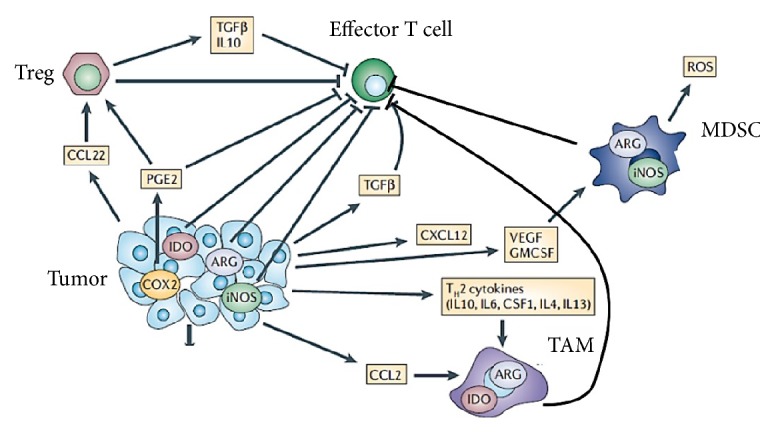
Metabolic interactions between tumor, stromal, and immune cells in the tumor microenvironment. Tumor and stromal cells express ARG, IDO, Cox2/Ptgs2, and iNOS/NOS2, which produce reactive oxygen species (ROS), chemokines, and Th2 cytokines that recruit Tregs, MDSC, and tumor-associated macrophages (TAM) to block effector T cell activation. Adapted from [84].

One of the primary mechanisms associated with cancer progression is the coopting of immune tolerance to produce an immunologically permissive tumor microenvironment [73]. This can occur through several mechanisms associated with adaptive immunity, including expansion of tumor infiltrating regulatory T cells (Tregs), myeloid-derived suppressor cells (MDSC), and tumor-associated macrophages (TAM) [74, 75] (Figure 2). Tregs contribute to immune escape by activation of the programmed cell death protein-1 (PD-1) receptor through immune and tumor cell expression of its ligand, PD-L1 (not shown), which results in suppression of effector T cell function mediated by CD4^+^ helper T cells and CD8^+^ cytotoxic T cells. MDSC also differentiate into TAM with similar T cell inhibitory properties [76], a process driven by inflammatory Th2 cytokines, which ultimately leads to tumor progression. Although there are numerous studies of these pathways in immune tolerance, the role of PPAR*δ* in this process has not been examined in mammary tumor models. Nevertheless, a clue as to its functional role in adaptive immunity may be gleaned from studies in diabetic obese mice. In liver and adipose tissue, PPAR*δ* is required to maintain insulin sensitivity via Th2 cytokines, which promote M2 macrophage polarization [77, 78] that have the characteristics of TAMs, and promotes tolerance to “self” recognition [79] to prevent diabetes. This suggests that PPAR*δ* may play a similar role in tumorigenesis, but with a decidedly different outcome. As discussed in Section 2, PPAR*δ* regulates the inflammatory Saa1/2/3 and S100a8/9 pathways, which in tumor-bearing mice are associated with MDSC expansion [80] and metastasis [81]. Immune tolerance mediated by Tregs, MDSC, and TAM are dependent on PGE_2_ synthesis, reactive oxygen species generated by NADPH oxidase (NOX1), and tryptophan depletion by indoleamine 2,3 dioxygenase (IDO) [74] (Figure 2), all of which are under the transcriptional control of PPAR*δ*. MDSC and Treg infiltration of mammary tumors is dependent on PGE_2_ synthesis and IDO activation [82], and inhibition of CD8^+^ T cell activation via the PD-1/PD-L1 axis is dependent on mTOR activation [83], a pathway that is activated in MMTV-PPAR*δ* mice [31]. Since the transcriptions of ARG1, IDO2, inducible nitric oxide synthetase (NOS2), Ptgs2, Ptger4, and NOX1 are all regulated by the coactivators CEBPA/B, which also function in this capacity with PPAR*δ*, this suggests a mechanism whereby PPAR*δ* may control adaptive immunity metabolically within the tumor microenvironment. This conclusion is also consistent with our recent finding that Plac1, which is overexpressed in MMTV-PPAR*δ* mice, mediates immune tolerance in murine breast cancer cells by upregulating the expression of chemokines necessary for MDSC-mediated activation of Tregs (H. Yuan and R. I. Glazer, unpublished results). Thus, there is compelling evidence, although circumstantial in some instances, which suggests that PPAR*δ* through its ability to regulate metabolic and inflammatory gene expression acts as a rheostat to control autoimmunity in normal tissues and immune tolerance during tumorigenesis.

## 6. Conclusions

Both genetic and pharmacological manipulation of PPAR*δ* expression provide strong evidence for its role in regulating metabolism, inflammation, and immunity in a concerted fashion to ultimately impact mammary tumorigenesis. This conclusion suggests possible novel targets for drug development that may control this process and complement current approaches to develop immunotherapies for the treatment of cancer.
